# A Short Review on the Intranasal Delivery of Diazepam for Treating Acute Repetitive Seizures

**DOI:** 10.3390/pharmaceutics12121167

**Published:** 2020-11-30

**Authors:** Sai H. S. Boddu, Sneha Kumari

**Affiliations:** 1 Department of Pharmaceutical Sciences, College of Pharmacy and Health Sciences, Ajman University, Ajman P.O. Box 346, UAE; 2 Center of Medical and Bio-allied Health Sciences Research, Ajman University, Ajman P.O. Box 346, UAE; 3 Department of Pharmacy Practice, The University of Toledo, Health Science Campus, 3000 Arlington Ave, Toledo, OH 43614, USA; sneha.kumari@utoledo.edu

**Keywords:** acute repetitive seizures, diazepam, intranasal, rectal gel, rescue therapy

## Abstract

Benzodiazepines such as diazepam, lorazepam and midazolam remained the mainstay of treatment for acute repetitive seizures (ARS). The immediate care for ARS should often begin at home by a caregiver. This prevents the progression of ARS to prolonged seizures or status epilepticus. For a long time and despite social objections rectal diazepam gel remained only FDA-approved rescue medication. Intranasal administration of benzodiazepines is considered attractive and safe compared with rectal, buccal and sublingual routes. Intranasal delivery offers numerous advantages such as large absorptive surface area, bypass the first-pass metabolism and good patient acceptance as it is needle free and painless. Recent clinical studies have demonstrated that diazepam nasal spray (NRL-1; Valtoco^®^, Neurelis Inc.,San Diego, CA, USA) showed less pharmacokinetic variability and reliable bioavailability compared with the diazepam rectal gel. Diazepam nasal spray could be considered as a suitable alternative for treating seizure emergencies outside the hospital. This review summarizes the treatment options for ARS and findings from clinical studies involving intranasal diazepam for treating seizure emergencies.

## 1. Introduction

An epileptic seizure is a neurologic condition marked by the occurrence of sudden and unpredictable interruption of neurons in the brain [1]. Epilepsy is characterized by two or more unprovoked seizures occurring 24 h apart. Approximately, 80% of seizures are unpredictable and sporadic, while 20% of seizures have a cyclical recurrence at somewhat predictable time intervals of days or weeks. Rare seizures occur in ~1% and are instigated by specific precipitating events. Acute repetitive seizures (ARS) or seizure clusters are often observed in patients with epilepsy. The definition of ARS was proposed by the National Institutes of Health Epilepsy Advisory Committee in the mid-1990s. ARS is characterized by multiple seizures in less than 24 h for adults or in less than twelve hours for children. The duration of ARS may vary from a few minutes to several hours [2,3]. ARS arises due to unusual activities in the brain that may go unnoticed or may result in convulsions and unconsciousness due to uncontrolled body shakes. Epileptic seizures, including ARS, can develop at any stage and any age [4]. Children with recurrent seizures exhibit characteristic episodes of repetitive seizures distinctly different from their usual seizure behavior. The terms used to describe this phenomenon in the past include serial, cluster, recurrent, flurries, repetitive seizures, and crescendo seizures [1,5].

According to the Center for Disease Control and Prevention, about 3,000,000 adults and 470,000 children in the USA are suffering from epilepsy [6]. The World Health Organization estimates over 50,000,000 people worldwide with epilepsy, of which approximately 80% are from low- and middle-income countries [7]. Generally, ARS is more common in patients with extratemporal epilepsy, specifically the frontal lobe epilepsy [8]. Other causes such as mesial temporal sclerosis [9], focal cortical dysplasia and a remote history of central nervous system infection are also shown to be associated with ARS [5,10]. Intractable epilepsy is also considered as a major risk factor for ARS with high average seizure frequency [10]. In most cases ARS is triggered due to sleep deprivation, hormonal changes during menstruation, stress, missing or changing medications, fever or illness, and alcohol [5,9]. Although, the overall percentage of the population with ARS is relatively small, those affected with ARS are at risk of medical complications, including injury, progression into status epilepticus and reduced quality of life. Given the substantial risks with ARS, it is necessary to develop appropriate protocols for identification and management of this seizure phenomenon [11]. The immediate care for ARS should often begin at home, before an emergency visit to a clinic [12]. Thus, it is crucial to treat short-term seizure recurrence, such as in ARS, to prevent its progression to prolonged seizures or status epilepticus. This review intends to provide an overview of treatment options for ARS, while emphasizing on clinical studies on the delivery of diazepam via the intranasal route.

## 2. Treatment Options for ARS

Drugs used in the management of seizure emergencies should (i) exhibit a rapid onset of action within a few minutes, (ii) be potent to permit small dose volumes, (iii) have a wide therapeutic index, and (iv) have an intermediate duration of action for a few hours. The major target for antiseizure drugs is the GABA_A_ receptor (GABA_A_R), which are found in the limbic system of the brain. Benzodiazepines (BZDs) are considered as the treatment of choice for acute management of severe seizures in both adults and children, except in infants less than two weeks of age in whom phenobarbitone is preferred [13]. Benzodiazepines are known to be positive allosteric modulators of GABA_A_ receptors. These receptors are chloride-selective ligand-gated ion channels that are activated by GABA (one of the major inhibitory neurotransmitter in the brain). Binding of GABA is promoted when benzodiazepines bind to this receptor complex. This leads to an increase in conduction of chloride ions across the neuronal cell membrane resulting in hyperpolarizing of the neuronal cell membrane potential. Thus, there is a significant increase in the difference between resting and threshold potential leading to the reduction of neuron firing [14].

**Figure 1:**
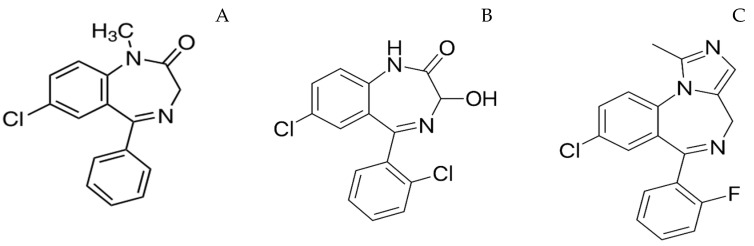
Structure of diazepam (**A**), lorazepam (**B**), midazolam (**C**).

Evidence suggests that repetitive seizures can produce lasting morphological cortical brain damage [15], and the treatment efficacy decreases with an increase in the duration of seizure [16]. Therefore, immediate care by a caregiver should begin with the onset of seizures to maximize the treatment efficacy. This approach is popularly known as rescue therapy. Parenteral administration (intravenous or intramuscular) of antiepileptic drugs by a medical personnel results in an average treatment initiation time of approximately 85 min. Intravenous (IV) administration of benzodiazepines is preferred in a hospital setting as it offers the fastest onset of action. Lorazepam, diazepam and midazolam are the most commonly administered IV preparations. These drugs have the same mechanism of action; however, the variation in their physicochemical and pharmacokinetic properties has clinical implications. For instance, the lipid solubility of benzodiazepines may vary by more than 3 times depending on the electronegative substituents. The octanol/buffer partition ratios of diazepam, lorazepam and midazolam at physiological pH are 309, 73 and 34, respectively [17]. The presence of methyl group in the benzodiazepine skeleton of diazepam contributes to its lipophilicity (Figure 1) [18]. The logP value of a drug determines its lipid solubility and the rate at which a drug diffuses across biological barriers, i.e., the greater a drug’s lipid solubility, the faster its absorption across the tissue membranes [19]. Diazepam results in higher brain concentrations with an onset of action of ~30 s as it is highly lipophilic compared to lorazepam and midazolam. However, its high lipid solubility results in rapid in vivo redistribution into peripheral tissues, leading to a decrease in brain concentrations. The clinical effectiveness of diazepam is ~20 min, and mostly a second drug is required to reduce the relapse rate if diazepam is used as a first-line drug [20]. On the other hand, lorazepam has a slightly lower onset of action (~2 min) and its duration of action is greater than 12 h due to less lipid solubility compared to diazepam. The pharmacokinetic parameters of these drugs following IV administration are presented in Table 1. Intravenous administration is not possible when a patient with epilepsy has breakthrough or cluster seizures outside of medical facilities such as home, day care, school or work. Moreover, IV administration is associated with side effects such cardiac dysrhythmias, hypotension and depression of the CNS. As a result, there is a lot of interest in alternate out-of-hospital rescue therapies given by other routes of administration, such as oral, rectal, nasal and buccal routes [21].

An ideal treatment option for ARS should be effective against a variety of seizure types, rapidly absorbed with a swift onset of action and consistent interpatient bioavailability, easily prepared and administered by anyone (including the patient during the intervals when consciousness is not altered), have a sustained duration of action with minimal side effects. In the 1990s, rectal diazepam gel (Diastat^®^, Valeant Pharmaceuticals North America LLC, Bridgewater, NJ, USA) was approved by the FDA to abort acute seizures. Rectal administration of diazepam results in a rapid absorption because of high vascularity. Diazepam after rectal administration showed approximately 80–90% bioavailability with an average peak concentration (C_max_) occurring 10–60 min following the drug administration (Table 2). After rectal administration, therapeutic concentration of diazepam in plasma (>200 ng/mL) was reached within five to ten minutes, similar to both IV and IM administration. Diastat^®^ has altered the seizure management plans in many patients; however, its use remained controversial mainly because of social and legal concerns [24]. Rectal lorazepam is effective in reducing seizures but shows high and variable bioavailability. Rectal administration is considered socially awkward in a non-private setting and caregivers administering rectal diazepam are worried about the risk of allegations of sexual abuse [25,26]. Also, it might be difficult to administer in patients confined to a wheelchair. Until the recent past, diazepam rectal gel is the only product available for use in immediate out of the hospital care for ARS in the United States at a dose of 0.2 to 0.5 mg/kg, depending on age and weight [27]. In 2012, buccal midazolam (Buccolam^®^, Viropharma, Downingtown, PA, USA) was approved by the European Medicines Agency (EMA) for the treatment of acute and prolonged convulsive seizures in 3 months old infants to adolescents (<18 years). Sublingual lorazepam showed efficacy in the management of serial seizures in children [28]. Expidet^®^ (Temesta, Pfizer, Germany) is an orodispersible tablet of lorazepam approved in Europe for the treatment of acute seizures in children [21]. However, buccal route is associated with limitations such as jaw clenching [29], hypersalivation, and uncontrollable swallowing resulting in variable pharmacodynamics [30]. A list of marketed products for treating ARS is presented in Table 3.

**Figure 2:**
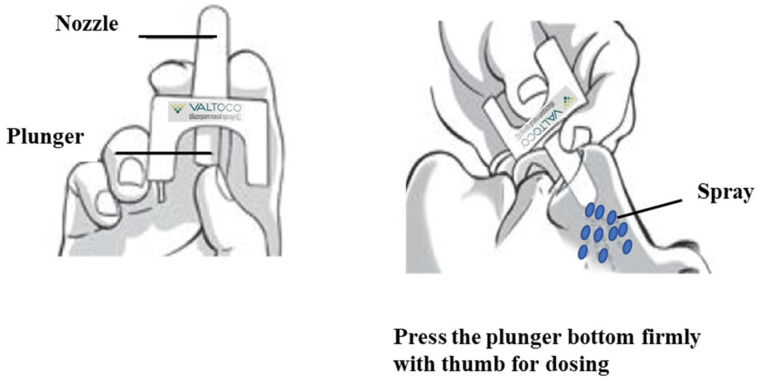
Application of Valtoco^®^ diazepam nasal spray. Image modified from Valtoco^®^ package insert.

Intranasal route is considered as an attractive alternative to the rectal and buccal routes of diazepam administration [35,36]. Development of intranasal formulations of benzodiazepines such as diazepam is challenging due to solubility and absorption issues arising from their nonaqueous nature [37]. Recently, FDA has approved intranasal formulations of diazepam (NRL-1; Valtoco^®^) and midazolam (USL261; Nayzilam^®^, Proximagen, LLC, Plymouth, MN, USA) for use by a caregiver outside of a medical setting for acute treatment of intermittent, stereotypic episodes of frequent seizure activity (i.e., seizure clusters, acute repetitive seizures) [38]. NRL-1 is formulated with vitamin E to enhance diazepam’s nonaqueous solubility and *n*-dodecyl β-d-maltoside (DDM, Intravail^®^ A3, Aegis Therapeutics, LLC, San Diego, CA, USA), a nonionic surfactant, as an absorption enhancer to promote the transmucosal bioavailability of diazepam [38,39]. Intravail^®^ excipients are designated as Generally Recognized as Safe (GRAS) substances for food applications [39]. Valtoco^®^ (5 mg and 10 mg doses) is generally administered as a single spray into one nostril, while higher doses of 15 mg and 20 mg doses requires two sprays, one spray into each nostril (Figure 2). To the best of our knowledge, very few review articles recapitulated the intranasal delivery approach for treating seizure emergencies and they are too generalized [37]. This review is mainly focused on the formulation approaches designed for intranasal delivery of diazepam for acute management of seizures.

## 3. Diazepam for Intranasal Administration

In the early 1980s, the nasal route was introduced as a promising alternative to the conventional delivery [40]. The nasal route is easily accessible and has a porous endothelial layer along with a highly vascularized epithelium to provide rapid absorption of drug compounds into the systemic circulation without first pass metabolism [41,42]. Other benefits of intranasal drug delivery include non-invasiveness, patient comfort and self-administration. The nasal cavity provides delivery of drugs ranging from small molecules to large macromolecules (proteins, peptides, hormones and vaccines) [43]. For information on formulation approaches for improved intranasal delivery of drugs the readers can refer to the review by Kapoor et al. [37]. Nasal route is favored to circumvent the obstacles of blood brain barrier (BBB) permitting direct drug delivery to the central nervous system (CNS). Nasally administered drugs can reach the brain via two pathways. In the direct pathway, drugs reach the brain via the olfactory and/trigeminal neuronal nerves, while in the indirect pathway, drugs are first absorbed into the systemic circulation and then reach the brain (graphical abstract) [37]. A study in New Zealand white rabbits and Sprague-Dawley rats showed that intranasal diazepam reached the brain predominantly via the indirect pathway with no significant direct nose-to-brain transport via olfactory epithelium and trigeminal neuronal nerves [44].

Diazepam, midazolam, lorazepam and clonazepam have been evaluated for intranasal administration as rescue therapy outside the hospital. Of these, midazolam exhibited a faster rate of absorption, while diazepam showed superior bioavailability and duration of action. The physicochemical properties of diazepam make it a good candidate for intranasal administration. Diazepam’s lipid solubility and potency are comparable to midazolam; however, diazepam has a substantially longer elimination half-life, which provides a longer duration of action as compared to midazolam [45]. Following intranasal administration, midazolam exhibits faster rate of absorption, but lower and more variable absorption, and a shorter elimination half-life as compared to intranasal diazepam [22,46]. Lorazepam is long acting (up to 72 h), with less risk of seizure recurrence. As compared with diazepam and midazolam, lorazepam has a slower redistribution from the brain due to its lower lipid solubility, resulting in a longer half-life. These properties make IV lorazepam a good choice for treating status epilepticus. In contrast, the lower lipid solubility of lorazepam makes it a less optimal candidate for intranasal administration due to slower rate of absorption and onset of action than midazolam and diazepam [47]. In a study, the efficacy of lorazepam administered via buccal, intranasal or IV routes were compared in Malawian children with acute seizures. This study concluded that lorazepam administered through intranasal and buccal routes was less effective compared to IV lorazepam [48]. Overall, intranasal diazepam has been considered as an attractive treatment option in ARS compared to other benzodiazepines.

Several studies have investigated the efficacy of intranasal diazepam in comparison with IV and rectal administration both in animals and humans. These studies have been highlighted in Table 4. In this section we shall focus mainly on the safety and efficacy studies of intranasal diazepam conducted in humans. The first clinical trial of intranasal diazepam in comparison with IV administration was conducted by Gizurarson et al. Intranasal diazepam was prepared in 5% glycofurol in polyethylene glycol 200. In this open crossover clinical trial, 2 mg diazepam was administered in nine healthy volunteers both intranasally and intravenously. The results showed that the bioavailability was 50.4 ± 23.3%, and the peak concentration was achieved after 18 ± 11 min following intranasal administration. The pharmacodynamic effect of diazepam was observed after ~5 min; however, IV administration failed to generate a strong measurable effect at such a low dose. Despite the transient nasal discomfort in subjects, this study concluded that intranasal diazepam could be an alternative to intranasal and IV administration for acute seizures [49]. A follow-up study by the same group assessed the electroencephalographic (EEG) effects, blood concentrations, vehicle irritation and dose-effect relationships of intranasal diazepam (dissolved in polyethylene glycol 300). Intranasal doses of 4 and 7 mg diazepam were compared with 5 mg IV dose in seven healthy volunteers. The relative bioavailability of 4 and 7 mg doses of intranasal diazepam was 45 and 42% with a C_max_ of 99 and 179 ng/mL and t_max_ of 18 and 42 min, respectively. The bioavailability of 4 and 7 mg intranasal formulations were found to be almost similar, 45% and 42%, respectively. EEG effects of the highest nasal dose (7 mg) and the intravenous dose were found to be similar, whereas the nasal dose of 4 mg produced a lower effect. This study pointed out that the lipophilic nature of diazepam promotes its distribution into the fatty tissue of the brain as the plasma concentration of the drug falls, resulting in a sustained pharmacodynamic effect. Nevertheless, this study did not choose to evaluate the existence of the direct nose-to-brain transport of diazepam. The authors concluded that PEG300 as a solubilizer of diazepam was found to be effective in the acute treatment of epilepsy [50].

Ivaturi and his colleagues studied the bioavailability, dose proportionality and tolerability of a supersaturated intranasal diazepam solubilized in a glycofurol-water cosolvent system in eight healthy volunteers [51]. A dose of 5 and 10 mg intranasal diazepam of the investigational formulation (supersaturated solution containing 40 mg/mL of diazepam in glycofurol and water) was compared with a 5 mg dose of IV diazepam. The median t_max_ were 20 and 30 min and the mean C_max_ were 134.3± 62 and 247.6 ± 6 1 ng/mL for 5 and 10 mg intranasal doses with an estimated bioavailability of 75% for both doses. This study reported reasonable bioavailability from intranasal diazepam with less tolerability as volunteers reported of discomfort after intranasal administration. This was attributed to the use of glycofurol in the intranasal formulation. In a subsequent study the same group assessed the pharmacokinetics and tolerability of investigational diazepam formulation and a parenteral midazolam following intranasal administration for treatment of seizure emergencies. Subjects received 5 mg of diazepam and midazolam via both IV and intranasal routes in a four-way, randomized crossover trial. Both formulations exhibited rapid drug absorption following intranasal administration with temporary discomfort. The C_max_ and T_max_ values for intranasal midazolam and diazepam were 62.8 ng/mL and 21.6 min vs 179.2 ng/mL and 28.8 min, respectively. Diazepam showed an extended duration of action due to a longer drug half-life. This study supported the usefulness of intranasal diazepam for treating seizure emergencies and emphasized on more research to understand the safety, efficacy and pharmacokinetics of intranasal diazepam in large patient populations [45].

In a follow-up study the pharmacokinetics of newly designed, better tolerating intranasal formulations and rectal gel of diazepam were compared. Nasal formulations such as 10 mg diazepam nasal formulation (Nas-A), 10 mg diazepam nasal formulation (Nas-B1) and 13.4 mg diazepam nasal formulation (Nas-B2) were compared with 10 mg rectal diazepam. While the components of nasal formulations were not disclosed, the authors mentioned that excipients used in marketed ophthalmic preparation were utilized with a considerably higher aqueous component. The data from this study suggested that the absorption profile from the nasal spray and rectal gel are very similar. T_max_ attained was between 30 to 60 min post dose and the concentration of diazepam was above threshold for up to 12 h. The maximum concentrations of intranasal formulations were in the range of 150–190 ng/mL. No clear advantage in pharmacokinetics was observed for either Nas-A or Nas-B, and their relative bioavailability was in the range of 70–90%. Nasal formulation was found to be comparable to rectal gel in bioavailability. Incorporation of GRAS approved excipients in the nasal formulation resulted in better tolerability. This made intranasal administration of diazepam a viable alternative. This study also noted a wide intra-individual variability regardless of the route of administration [63]. Further study of the newly designed, better tolerable intranasal formulations were compared with a 5 mg IV dose of commercially available diazepam injectable in 24 healthy volunteers. Intranasal diazepam solution resulted in an absolute bioavailability of 97% with T_max_ value of 1.5 h and half-life of approximately 49 h. This study concluded that the tested intranasal solution of diazepam resulted in high bioavailability, good tolerability, and reasonable variability [32]. This study unlike the previous studies did not report any nasal irritation; however, the issue of intersubject variability remained.

Herbert et al. in collaboration with Acorda Therapeutics (New York, NY, USA) developed a nasal spray of diazepam and evaluated its tolerability and bioavailability relative to rectal diazepam in 24 healthy adults. The nasal spray of diazepam was prepared using inactive ingredients such as methyl laurate, diethylene glycol monoethyl ether, N-methyl-2-pyrrolidone, propylene glycol monocaprylate, ethanol and water. Two intranasal doses of 5 mg and 20 mg were used in dose proportionality studies. The mean C_max_ values for 20 mg intranasal diazepam and rectal diazepam were 378 ± 106 and 328 ± 152 ng/mL with T_max_ values of 1.0 and 1.5 h, respectively. Subjects administered intranasal and rectal gel formulations experienced nasal and rectal leakage, respectively. This study concluded that a single-dose of 20 mg intranasal diazepam was comparable to rectal diazepam in terms of tolerability and bioavailability. Non treatment-emergent adverse events such as headache was reported in a few volunteers administered with 20 mg intranasal diazepam [66]. The same group further examined the dosing feasibility and tolerability of intranasal diazepam (single dose of 0.2 mg/kg) in adults with epilepsy. This pharmacokinetic study showed that a single dose can be effectively administered to adult patients either during or immediately after seizures. The differences in breathing patterns, body position or seizure type had no impact on the effectiveness of spray since the C_max_ and AUC values were comparable in different types of seizures. Even though a few patients faced nasal discharge immediately after dosing, the drug was rapidly absorbed. The data from 30 subjects showed that diazepam levels reached a mean concentration of 158 ± 57.2 ng/mL by 15 min following dose administration and gradually reduced to 77 ng/mL by 12 h. Based on the pharmacokinetic parameters, this study concluded that the discharge did not have a significant effect on the loss of diazepam. The plasma concentration of diazepam was reported to be sustained over 12 h and was within the reported range to display anticonvulsant effects. This study concluded that the intranasal diazepam has the potential to provide patients with a treatment option that offers greater freedom of movement beyond their local communities and emergency medical support, while addressing the issues of inconvenience and social embarrassment associated with rectal administration [65]. However, this study did not compare the efficacy their formulation against diazepam rectal gel in treating seizure clusters.

In 2016, Acorda Therapeutics Inc. published a press release reporting that Plumiaz nasal spray containing diazepam did not show good absorption across the lining of the nose in comparison with rectal diazepam or Diastat AcuDial [77]. Later in 2017, Neurelis Inc. (San Diego, CA, USA) announced that the US FDA has designated NRL-1 (intranasal diazepam) on a Fast Track Development program for the management of selected, refractory patients with epilepsy, on a stable regimen of anti-epileptic drugs (AEDs), who require intermittent use of diazepam to control bouts of increased seizures—also referred to as acute repetitive or cluster seizures [78]. NRL-1 (intranasal diazepam) is a nasal spray developed for the management of adult and pediatric patients that face ARS. In clinical trials, NRL-1 has demonstrated low variability, high bioavailability and was well-tolerated. This proprietary formulation of diazepam (NRL-1, Valtoco^®^) has been granted Orphan Drug and Fast Track Designations by the FDA.

## 4. Conclusions

Epilepsy encompasses many syndromes and subsyndromes with a vast array of causes, effects and different underlying mechanisms. However, there are common mechanisms of seizure generation and propagation that can be successfully targeted by a single pharmacological agent. For instance, benzodiazepines are broadly effective across all seizure types and epilepsy syndromes, even though there are patients who are resistant to these drugs. Government organizations worldwide are trying to curb overall healthcare expenditure by encouraging patient-treatment through outpatient care models, such as clinic and home healthcare. Due to this reason, ARS market is expected to surge in the near future. The use of benzodiazepines as rescue medication in the management of ARS can help reduce healthcare-related expenses in patients due to decreased emergency room visits. Once delivered into the central nervous system, benzodiazepines are shown to be effective against a wide range of seizures with a rapid onset of action and thus can reduce emergency room visits. Intranasal diazepam has found a niche in alternate out-of-hospital rescue therapies of ARS, as several studies have demonstrated its undeniable positive effect on ARS. Diazepam nasal spray demonstrated an acceptable safety profile with less variation in the bioavailability compared with the rectal route. On the basis of pharmacokinetic results, tolerability and safety, diazepam nasal spray has the potential to be a game-changing treatment in ARS.

Current understanding of the mechanisms underlying epileptogenesis, seizure generation and seizure spread is advancing at an unprecedented pace, paving the way to the design and identify compounds that have the potential of improving the clinical outcomes in a staggering way. Major breakthroughs are being made in understanding the molecular deficits underlying many of these syndromes, including epileptic encephalopathies of childhood, making it possible to rationally design highly efficacious compounds precisely targeting the underlying etiological mechanisms. These modern advances will enable the investigation of antiepileptic drug pharmacology and facilitate the commercialization of new drug molecules by improving drug stability, solubility, decreasing dose frequency, and expanding routes of administration to address the unmet clinical needs. Further, technological advances in drug delivery allow researchers to administer drugs, control the rate of absorption and target them to a specific site. There is also a growing interest in seizure prediction using EEG recordings. The ability to predict seizures prior to onset, could transform the current mode of therapy to administering of medication well before the seizure occurs. Collaborative efforts among pharmaceutical scientists, physicians, and engineers are the need of the hour for invention of new technologies to improve the management of epilepsy.

## Figures and Tables

**Table 1:** Pharmacokinetic parameters of diazepam, midazolam and lorazepam following intravenous administration [22,23].

Drug	Volume of Distribution (V_d_), L/kg	Clearance (L/h/Kg)	Distribution Half-Life (t_1/2α_) (min)	Elimination Half-Life (t_1/2β_) (min)	Onset of Action (min)	Duration of Action
Diazepam	0.89 ± 0.18	0.0388 ± 0.015	1.9–13.3	32.9 ± 8.8	1–3	<2 h
Midazolam	0.80 ± 0.19	0.42 ± 0.17	18.6 ± 14.4	2.4 ± 0.8	~2	3–4 h
Lorazepam	1.14 ± 0.03	0.063 ± 0.009	<11	14.3 ± 2.5	1–3	<72 h

**Table 2:** Pharmacokinetic parameters of diazepam following various routes of administration [31,32,33].

Route of Administration	Onset of Action (min)	Peak Plasma Levels (min)	Bioavailability
Oral	15–60	30–90	100%
IM	15–30	30–60	60%
Rectal	5–10	10–45	80–90%
Intranasal	<5	>60	97%

**Table 3:** List of marketed products for acute repetitive seizures.

Drug	Brand Name	Route	Excipients
**Diazepam**	Valium^®^	Intravenous	40% propylene glycol, 10% ethyl alcohol; 5% Na benzoate and, benzoic acid as buffers, and 1.5% benzyl alcohol as a preservative [34].
**Diazepam**	Diastat^®^	Rectal	Propylene glycol, ethyl alcohol (10%), hydroxypropyl methylcellulose, sodium benzoate, benzyl alcohol (1.5%), benzoic acid and water.
**Diazepam**	Valtoco^®^ (Available doses: 5 mg, 10 mg, 15 mg)	Intranasal	Benzyl alcohol (10.5 mg per 0.1 mL), dehydrated alcohol, n-dodecyl beta-d-maltoside, and vitamin E.
**Lorazepam**	Temesta^®^, solution for injection	Intravenous	Macrogol 400, benzyl alcohol 21 mg/ml, propylene glycol.
**Lorazepam**	Temesta Expidet^®^	Orodispersible	Gelatin, mannitol
**Midazolam**	Midazolam injection, USP	Intravenous	0.8% sodium chloride and 0.01% edetate disodium, with 1% benzyl alcohol as preservative; the pH is adjusted to 3 to 3.6 with hydrochloric acid and, if necessary, sodium hydroxide
**Midazolam**	Seizalam™	Intramuscular	1% benzyl alcohol as preservative, 0.01% edetate disodium, and 0.8% sodium chloride, pH is adjusted to ~3.
**Midazolam**	Nayzilam^®^	Intranasal	Ethanol, PEG-6 methyl ether, polyethylene glycol 400, propylene glycol and purified water.
**Midazolam hydrochloride**	Buccolam^®^	Oromucosal solution	Sodium chloride, water for injections, hydrochloric acid (for pH adjustment and conversion of midazolam to the hydrochloride salt), sodium hydroxide (for pH adjustment
**Midazolam maleate**	Epistatus^®^	Oromucosal solution	Ethanol, saccharin sodium, glycerol purified water, sodium hydroxide (for pH adjustment), liquid maltitol

**Table 4:** Preclinical and clinical pharmacokinetic studies related to the use of intranasal diazepam.

Formulations/Routes of Administration	Study Design	Subjects	Results	Conclusions	Ref.
Intranasal diazepam (10 mg) and lorazepam (4 mg) formulated using non-ionic surfactant (Cremophor EL)	Crossover trial	Healthy adults	Diazepam: Bioavailability = 84% and 72%, T_max_ = 1 h, C_max_ = 175 ng/mL. Peak concentration following intranasal administration was 27% to that of IV administration. Lorazepam: Bioavailability: 35–63%, T_max_ = 0.5 to 4 h, C_max_ = 18.7 ± 5.9 ng/mL, C_max_: 33–94% to that following IV administration.	Intranasal administration of diazepam and lorazepam would have limited potential in the acute treatment of seizures.	[52]
Intranasal diazepam in a mixture of 5% glycofurol and polyethylene glycol 200 versus commercial IV diazepam (Stesolid^®^ Dumex-Alpharma), 2 mg dose	Open crossover trail	Healthy students	Intranasal diazepam: C_max_= 39 ± 17 ng/mL, t_max_ = 18 ± 11 min, t_1/2_ = 17.8 ± 15.5, AUC_0–30min_ = 1095 ± 412 ng.min/mL. Intravenous diazepam: t_1/2_ = 14.4 ± 7.0, AUC_0–30min_ = 2972 ± 980 ng.min/mL.	Intranasal diazepam could be an alternative to IV and rectal administration for treating acute seizures	[49]
Intranasal diazepam in polyethylene glycol 300 (4 mg and 7 mg dose) versus Stesolid Novum^®^ intravenous diazepam (5 mg dose)	Double-blind, randomized, crossover design	Healthy volunteers	Mean differences between before and after drug administration values of P300-N100 amplitude differences were −0.9 (6.5, 4.7), −6.4 (−10.1, −2,7), −8.6 (−11.4, −5.8) and −9.6 (−12.1, −7.1) for placebo, 4 mg intranasal, 7 mg intranasal and 5 mg diazepam preparations, respectively. 4 and 7 mg intranasal diazepam formulations showed bioavailabilities of 45% and 42%, respectively.	Based on the electroencephalographic effects and blood concentration data, PEG300 may be used to deliver effective nasal dose of diazepam for the acute treatment of epilepsy	[50]
Intranasal diazepam in ten vehicles of different polarity to achieve t_max_ ≤ 5 min	-	Rabbits	Pure glycofurol 75, tetraethyleneglycol, poly(ethylene glycol) 200 and 30% glycofurol in tetraethyleneglycol showed very rapid pharmacodynamic response (1.5–3.5 min) compared to more polar liquids	Water-free low-molecular-weight glycols might be used as an alternative to IV injection for acute situations.	[53]
Intranasal diazepam versus IV diazepam (0.5 mg/kg)	Crossover design	Healthy adult greyhounds	IV: C_peak_: 1316 ± 216 µg/L, T_peak_ was ≤ 3 min Intranasal: C_peak_: 448 ± 41 µg/L), T_peak_: 4.5 ± 1.5 min, Bioavailability: 80 ± 9%	Plasma concentrations exceed 300 µg/L (therapeutic concentration). Intranasal diazepam may be useful for treatment of seizures in dogs in place of IV administration	[54]
Intranasal ethyl laurate-based microemulsion systems of diazepam (1–2 mg/kg) and comparison with IV administration (1 mg/kg).	-	Rabbits	Tween 80–23.3%, propylene glycol–23.3% ethanol–15% H_2_O at 2 mg/kg dose resulted in rapid-onset of action (2–3 min) of diazepam with 50% bioavailability.	Ethyl laurate-based microemulsion of diazepam may be useful in the treatment of status epilepticus.	[55]
Diazepam intranasal (7 mg) versus diazepam intravenous (3 mg). Results were compared with rabbit and human data	Crossover design	Sheep	Mean nasal bioavailability, t_max_ and C_max_ were 15 ± 8%, 5 ± 3 min and 934 ± 593 ng/mL, respectively. Bioavailability of diazepam in sheep was lower than rabbit (54%, *p* < 0.001) and man (34%, *p* < 0.05).	Correlation of bioavailability (rate and the extent of absorption) was not optimal between sheep, man and rabbit.	[56]
Supersaturated solution of diazepam in glycofurol/water for intranasal administration	-	MDCK epithelial cells as a nasal mucosa model	Steady-state flux of diazepam was obtained across MDCK epithelial cell monolayers from supersaturated solutions, which increased proportionally with increasing degree of saturation	Supersaturated diazepam solutions may be used for intranasal delivery	[57]
Diazepam was intravenously (1 mg/kg) or intranasally (2 mg/kg) administered to rats and rabbits	-	New Zealand white rabbits and Sprague–Dawley rats	Rats: T_max_ = 5 min in rats, AUC_brain_/AUC_plasma_ ratios after IV (3.03 ± 0.07) and intranasal (3.00 ± 0.32) administration were nearly identical. Bioavailability in rat plasma (68.4%) and brain (67.7%) Rabbits: T_max_ = 10 min, AUC_brain_/AUC_plasma_ ratios after intranasal administration (3.77 ± 0.17) were slightly lower than from IV administration (4.23 ± 0.08). Bioavailability in rabbit plasma (51.6%) and brain (45.9%)	No significant nose-to-brain transport (via olfactory epithelium) of diazepam was observed. Diazepam was mostly transported acorss the blood–brain barrier after intranasal administration.	[44]
Intranasal diazepam microemulsion	-	Bufo gargarizans	Miglyol 812 (8.0%), Tween 80 (21.3%), PEG400 (10.7%) and water (60.0%) containing microemulsion of diazepam showed only slight nasal ciliotoxicity	Microemulsions of Miglyol 812-Tween80-PEG400-water system with diazepam could be used for intranasal administration	[58]
5 mg of diazepam and midazolam via both intranasal and IV routes	Four-way, randomized crossover trial.	Healthy adult volunteers	Diazepam: C_max_ = 179.2° ng/mL, T_max_ = 28.8 min Midazolam: C_max_ = 62.8° ng/mL and T_max_ 21.6 min. Intranasal administration resulted in rapid absorption with transient discomfort. Diazepam had a longer half-life, with an extended duration of action	Diazepam and midazolam were rapidly absorbed following intranasal administration with transient discomfort.	[45]
Intranasal formulation of diazepam (5 mg and 10 mg) in a glycofurol–water cosolvent system was investigated	Randomized, single-blind, three-way crossover	Healthy volunteers	The estimated bioavailability was 75% with pain and tolerability scores around 2–2.3 and 4.4–4.7 following administration of 5 and 10 mg doses, respectively	Intranasal diazepam provided a reasonable bioavailability, but was not well tolerated	[51]
Alcohol-free microemulsion system for intranasal delivery of diazepam or midazolam (2.5% by weight)	Randomized cross-over design	Rabbits	Diazepam: C_max_ = 8.40 ± 3.00, Absolute bioavailability = 33.45 ± 12.36%, t_max_ = 18.33 ± 23.09 min Midazolam: C_max_ = 46.62 ±17.38, t_max_ = 9.25 ± 6.75 min, Absolute bioavailability = 35.19 ±11.83%	Midazolam and diazepam microemulsion system could achieve rapid-onset of action following intranasal administration	[59]
Pharmacokinetics of diazepam following IV administration versus administration as intranasal drop versus atomized nasal administration	Randomized block design	Dogs	Mean diazepam concentrations following intranasal administration reached >300 ng/mL within 5 min in both groups. Diazepam bioavailability after intranasal drop and atomized nasal administration was 42% and 41%, respectively	Intranasal administration yielded rapid anticonvulsant concentrations of diazepam in dogs	[60]
Effect of l-menthol on absorption of intranasal diazepam	-	Mice	The effect of diazepam via intranasal administration was strengthened in the presence of l-menthol	Intranasal diazepam with l-menthol may result in sedative-hypnotic action and control epileptic seizures	[61]
Effect of menthol as a penetration enhancer on the absorption intranasal diazepam		Rabbit	At 0.2%, menthol increased the absorption of diazepam [k = (0.4424 ± 0.0023)/h] with quick absorption [t_1/2_ =(0.32 ± 0.07)h]	0.2% menthol helped in the intranasal absorption of diazepam through passive diffusion	[62]
Tolerability and pharmacokinetics of two intranasal diazepam formulations were compared with rectal gel (Diastat^®^)	Double blind, 4-period, 4-way crossover study	Healthy volunteers	Mean C_max_ (± SD) was 181.8 ± 84.16, 151.3 ± 108.1 and 180.7 ± 82.1 ng/mL for Nas-A 10 mg, Nas-B 10 mg and Nas-B 13.4 mg respectively; while C_max_ for the rectal gel was 160.9 ± 109.4 ng/mL. Median t_max_ was 0.75 h for all treatments. Intranasal formulations were well tolerated and exhibited relatively rapid but variable absorption with bioavailability of 70–90% compared to diazepam rectal gel	Intranasal diazepam could be an alternative to rectal diazepam	[63]
Dose proportionality of 5 mg and 20 mg of intranasal diazepam formulations. Relative bioavailability of 20 mg intranasal diazepam versus 20 mg rectal gel	Phase 1, single-center, randomized, open-label, three-period crossover study	Healthy subjects	Intranasal diazepam solutions (5 and 20 mg) showed dose proportionality with median time to C_max_ of 1 h. Administration of a single dose of 20 mg intranasal diazepam resulted in similar plasma concentrations of diazepam and metabolite concentration, with less variability than with 20 mg rectal gel	Diazepam nasal solution (20 mg) showed comparable bioavailability as 20 mg rectal gel	[64]
Diazepam nasal spray (0.2 mg/kg)	Open-label study		T_max_ of diazepam was 45 min with comparable dose-normalized mean C_max_ and AUC_0–12_ values of diazepam among patients regardless of the timing of administration in relation to seizure.	Diazepam nasal spray could be used during the convulsive phase of tonic-clonic seizures or in the postictal periods following tonic-clonic or other seizure types.	[65]
Intranasal diazepam formulation versus an equivalent dose of rectal diazepam (20 mg)	Phase 1, open-label, 3-period crossover study.	Healthy adults	Mean C_max_ values of diazepam nasal spray and rectal gel were found to be 378 ± 106 and 328 ± 152 ng/mL, achieved at 1.0 and 1.5 h, respectively. Both intranasal and rectal diazepam were well tolerated with mild to moderate adverse events.	Single-dose of 20 mg diazepam nasal spray is tolerable and comparable in bioavailability to that of diazepam rectal gel.	[66]
Supersaturated diazepam solution using a prodrug/enzyme system (Avizafone, a peptide prodrug of diazepam, delivered with—Aspergillus oryzae protease)	-	Madin-Darby canine kidney II-wild type	Prodrug-protease mixtures upon apical exposure onto MDCKII-wt monolayers showed 2–17.6-fold higher diazepam flux (S = 1.3–15.3) compared to saturated diazepam (S = 0.7).	Intranasal avizafone-protease system with diazepam may provide rapid delivery.	[67]
Effectiveness of intranasal diazepam as an effective alternative to IV diazepam based on the medical records	Retrospective study	Stroke patients presenting with status epilepticus.	Intranasal diazepam was administered 9 times faster compared to IV diazepam resulting in about 3-fold reduction in the time to seizure activity termination following arrival at the hospital (3 min vs 9.5 min in the IV group, *p* = 0.030)	Intranasal diazepam could be a safe, quick and easier alternative to intravenous administration.	[68]
Diazepam-loaded poly(lactic-co-glycolic acid) nanoparticles	-	Rats	Gamma scintigraphy using technetium-99m-labeled (99mTc) showed a higher uptake of diazepam from nanoparticles compared to diazepam suspension in Sprague-Dawley rats.	PLGA nanoparticles of diazepam could be used in the treatment of status epilepticus	[69]
Mucoadhesive microemulsions of diazepam for intranasal administration versus Calmpose (i.v) and microemulsions	-	Rats	Diazepam microemulsion composed of oleic acid (5%), surfactant mixture (50%), water (45%), and chitosan (0.5%) showed significantly high flux of 846.96 ± 34 µg/cm^2^/h and AUC_brain_ = 1206.49 ± 145.8 compared to Calmpose (i.v) and microemulsion.	Mucoadhesive microemulsions showed higher absorption compared to IV administration	[70]
Coadministration of a hydrophilic diazepam prodrug (avizafone) and converting enzyme, human aminopeptidase B	-	Rats	Single doses of intranasal avizafone equivalent to 0.500, 1.00, and 1.50 mg/kg of diazepam resulted in 77.8% ± 6.0%, 112% ± 10%, and 114% ± 7% bioavailability with C_max_ plasma concentrations 71.5 ± 9.3, 388 ± 31, and 355 ± 187 ng/mL; and t_max_ of 5, 8, and 5 min for each dose level, respectively.	Rapid and complete absorption by co-administering avizafone with aminopeptidase B	[71]
1 dose period (5, 10, and 20 mg) followed by a 2-dose period (2 × 10 mg) with a minimum 28-day washout	Phase 1, open-label, randomized, crossover study	Healthy adult volunteers	Plasma concentration-time profiles showed similar patterns in a dose-dependent manner. The C_max_ values of diazepam were 85.6, 133.6, and 235.3 ng/mL for 5-, 10-, and 20-mg doses, respectively. Dose-normalized AUC_0–∞_ values were comparable between the 2 × 10-mg and single 10-mg doses.	NRL-1 could be a potential therapy for managing seizure emergencies.	[38]
Valtoco™ (NRL-1; diazepam nasal spray) formulated with Intravail^®^ A3	Open-label study	Patients with epilepsy	Pharmacokinetic parameters in ictal/peri-ictal and inter-ictal conditions were similar (t_max_: 3.31 ± 2.10 vs. 2.79 ± 1.89; C_max_: 156 ± 17 vs. 179 ± 18 ng/mL; AUC: 518 ± 30 vs. 566 ± 33 hr·ng/mL, respectively)	Valtoco™ showed a good safety profile in patients with epilepsy	[72]
Bioavailability and tolerability of intranasal diazepam containing Intravail^®^ vs diazepam rectal gel	Phase 1, open-label, randomized, single-dose, crossover study	Healthy adult subjects	T_max_ was similar for diazepam nasal spray and rectal gel, which were slower than oral diazepam in fasted individuals	Intravail^®^ provided therapeutic levels of diazepam comparable to rectal diazepam with no damage to the nasal mucosa	[73]
Tolerability of NRL-1 (Valtoco^®^, diazepam nasal spray formulated with Intravail A3) and adverse events in patients	Open-label, Phase 3 study	Adults and children/adolescents with epilepsy	Of the 57 subjects, 17 subjects (29.8%) reported treatment emergent adverse events (TEAEs) with no treatment discontinuation. Treatment-related TEAEs were observed in 8 subjects (14%). Dysgeusia was reported in 3 subjects (5.3%) and nasal discomfort in 2 subjects)	NRL-1 demonstrated an acceptable safety/tolerability profile	[74]
Long-term safety and tolerability of NRL-1 (Valtoco^®^, diazepam nasal spray formulated with Intravail A3). A dose of 5, 10, 15, or 20 mg was administered based on patient weight	Phase 3, open-label, study	Patients (including adults and children/adolescents	Out of 132 enrolled subjects, NRL-1 was used moderately in 65 (49.2%) and frequently in 67 (50.8%) patients. Overall, 91 patients (68.9%) had TEAEs	Repeat dosing of NRL-1 showed an acceptable safety/tolerability profile similar to diazepam administered via other routes	[75]
Type of dosing errors and extent as a substitution for the ability of patients/caregivers to properly administer NRL-1	Phase 3, open-label, study	Pediatric and adult patients with epilepsy	Patients/caregivers reported 31 dosing errors in 23 patients (1.2% of the administered 2486 doses). 80.6% of these errors were associated with doses requiring spray into both nostrils and 4 patients had multiple errors. Partial dosing errors were 48.4%, improper dosing errors were 12.9%, mechanical dosing time were 9.7% and 29.0% were other/unknown errors.	Most errors occurred when dose administration is required into both nostrils	[76]
